# Rethinking the role of cartilage loss: the influence of intra- and extra-articular factors on symptoms in advanced knee osteoarthritis

**DOI:** 10.1302/2633-1462.612.BJO-2025-0036.R1

**Published:** 2025-12-16

**Authors:** Luca Bianco Prevot, Alessandro Bensa, Pietro S. Randelli, Giuseppe Filardo

**Affiliations:** 1 Residency Program in Orthopedics and Traumatology, University of Milan, Milan, Italy; 2 IRCCS Ospedale Galeazzi - S. Ambrogio, Milan, Italy; 3 Service of Orthopaedics and Traumatology, Department of Surgery, Ente Ospedaliero Cantonale (EOC), Lugano, Switzerland; 4 Università della Svizzera Italiana, Faculty of Biomedical Sciences, Lugano, Switzerland; 5 First Orthopedics Clinic, ASST Centro Specialistico Ortopedico Traumatologico Gaetano Pini-CTO, Milan, Italy; 6 Laboratory of Applied Biomechanics, Department of Biomedical Sciences for Health, University of Milan, Milan, Italy

**Keywords:** Knee, Osteoarthritis, Cartilage, knee osteoarthritis, cartilage loss, Western Ontario and McMaster Universities osteoarthritis index, tendinitis, BMI, bone marrow lesions (BMLs), knees, quadriceps, cartilage degeneration, subchondral bone

## Abstract

**Aims:**

Understanding the factors contributing to pain and function limitation in knee osteoarthritis (OA) is crucial to optimize the individual patient’s management. This study aimed to quantify the role of cartilage degeneration, as well as other intra- and extra-articular factors, in determining clinical symptoms in patients with advanced knee OA.

**Methods:**

Subjects were selected from the Osteoarthritis Initiative database based on the criteria: Kellgren-Lawrence (KL) grades 3 to 4 and baseline clinical and MRI data. The analyzed data were: demographic parameters, KL grade, subchondral bone without cartilage coverage, anterior knee pain due to patellar quadriceps tendinitis, effusion, anserine bursa tenderness, meniscal extrusion, Hoffa’s inflammation, bone marrow lesions (BMLs), visual analogue scale for pain, Western Ontario and McMaster Universities osteoarthritis index (WOMAC) total score, and WOMAC pain subscale.

**Results:**

The multivariate analysis on 233 knees demonstrated that VAS was influenced by the percentage of femoral subchondral bone without cartilage coverage (p < 0.001/η² = 0.058), patellar quadriceps tendinitis (p = 0.004/η² = 0.036), BMI (p = 0.013/η² = 0.027), age (p = 0.026/η² = 0.022), and anserine bursa tenderness (p = 0.033/η² = 0.020). However, the WOMAC total score was influenced by patellar quadriceps tendinitis (p < 0.001/η² = 0.114), BMI (p = 0.001/η² = 0.045), female sex (p = 0.016/η² = 0.025), and medial compartment BMLs (p = 0.015/η² = 0.029), but not by the extent of cartilage damage.

**Conclusion:**

The extent of cartilage degeneration influences the pain level, but is not the main factor driving the overall symptoms experienced in advanced knee OA. Other intra- and extra-articular factors, including patellar quadriceps tendinitis, anserine bursa tenderness, BMLs, and BMI have a greater impact on pain and functional impairment, and should be considered when choosing the most suitable treatment approach to manage knee OA patients.

Cite this article: *Bone Jt Open* 2025;6(12):1611–1618.

## Introduction

Knee osteoarthritis (OA) is a chronic degenerative disease and one of the leading causes of musculoskeletal pain and disability worldwide.^1^ Knee-related symptoms significantly affect the quality of life of the affected patients.^2^ While being a chronic and progressive disease, in clinical practice OA is not always detected in its early stages, and many patients present with advanced disease, such as Kellgren-Lawrence (KL) grades 3 and 4,^3^ thus requiring more complex management.^4,5^ To this regard, cartilage degeneration is widely recognized as a central feature of the disease and is often considered a key aspect in the treatment algorithm.^6^ However, the relationship and interplay between cartilage loss and clinical symptoms remains unclear, even in advanced OA.

Treatment indications in the clinical practice as well as inclusion criteria in the research setting largely rely on the cartilage degeneration level. However, several studies reported that some patients with severe OA, including those with KL grades 3 and 4, may be asymptomatic or experience only mild symptoms.^7^ This discrepancy highlights the complex and multifaceted nature of symptoms in knee OA, suggesting that they may arise from an interplay of various intra-articular and extra-articular factors.^8^ Despite advances in knee OA understanding, the extent of the contribution of cartilage degeneration to pain and function limitations, as well as their progression over time, remain incompletely understood as well as its interplay and role within those of specific joint structures.^9-11^ Their complex interaction makes it challenging to identify the primary driver of symptoms in individual patients. A deeper understanding of the factors contributing to pain and function limitation in knee OA is crucial to optimize the individual patient’s management, as identifying the dominant drivers could facilitate more personalized treatment strategies, potentially delaying the need for invasive and costly approaches like total knee arthroplasty (TKA) for specific patients who could instead benefit from targeted interventions addressing specific symptoms contributors.

This study aimed at quantifying the role of cartilage degeneration, as well as other intra- and extra-articular factors, in determining clinical symptoms, specifically pain and functional impairment, in patients affected by advanced knee OA.

## Methods

### Study design

The subjects included in this study were selected from the Osteoarthritis Initiative (OAI) database, a multicentre, prospective, observational research project comprising 4,796 participants. The data used in this study are publicly available on the OAI website.^12^ The OAI database provides extensive data on the enrolled patients, including clinical parameters, and radiological and MRI images, as well as questionnaires assessing pain and function, such as the Western Ontario and McMaster Universities osteoarthritis index (WOMAC)^13^ and the visual analogue scale (VAS) for pain.^14^ An algorithm was developed using Python v. 3.9 and the Pandas library to identify advanced knee OA patients meeting the following inclusion criteria within the OAI database: KL grades 3 and 4, and baseline MRI imaging.

For each patient, the following baseline data were collected: knee side, age, sex, BMI, KL grade, percentage of subchondral bone not covered by cartilage for both femur and tibia, WOMAC total score (0 to 96), WOMAC pain subscale (0 to 20), VAS pain score (0 to 10), patellar quadriceps tendinitis (0= absent, 1= present), presence of effusion (positive bulge sign: 0 = absent, 1 = present), tenderness of the anserine bursa (0= absent, 1= present), medial and lateral meniscus extrusion (0 = < 2 mm, 1 = between 2 mm and 5 mm, 2 = > 5 mm), Hoffa body synovitis (0 = absent, 1 = mild, 2 = moderate, and 3 = severe), and bone marrow lesions (BMLs) in the femur and tibia, evaluated using the MRI Osteoarthritis Knee Score (MOAKS) score (0 to 45).^15^

### Patient characteristics

Using the search process and criteria outlined above, a total of 233 knees were identified from the OAI database for inclusion in the analysis. The detailed characteristics of the included patients are presented in Table I.

**Table I.** Characteristics of the included patents (n = 233).

Variable	Data	Variable	Data
**Mean age, yrs (SD)**	63.6 (9.3)	**Hoffa synovitis, %**	
**Sex, n (%)**		Normal	48.3
Male	101 (43.3)	Mild	37.5
Female	132 (56.7)	Moderate	11.9
**Mean BMI, kg/m** ^**2**^ **(SD)**	30.1 (5.1)	Severe	2.3
**Anserine bursa tenderness, %**	33	**Mean score (SD)**	
**Patellar quadriceps tendinitis, %**	19.3	WOMAC total, 0 to 96	22.5 (16.1)
**Medial meniscus extrusion, mm, %**		WOMAC pain subscale, 0 to 20	4.6 (3.6)
Normal	19.80	VAS pain score, 0 to 10	4.3 (2.6)
< 2	18	**Mean bone denuded from cartilage (SD)**	
2 to 5	45	Total	24.9 (27.8)
> 5	17.2	Femur	12.8 (16.8)
**Lateral meniscus extrusion, mm, %**		Tibia	12.1 (14.4)
Normal	80	**Kellgren-Lawrence grade, %**	
< 2	4	3	86.3
2 to 5	12	4	13.7
> 5	4	Mean total BML score, 0 to 45 (SD)	5.8 (3.3)

BML, bone marrow lesion; WOMAC, Western Ontario and McMaster Universities osteoarthritis index.

### Patient evaluation

Knee pain and function were evaluated at baseline using the WOMAC total score, WOMAC pain subscale, and VAS score. The WOMAC index is the most common used clinical tools for evaluating patients with knee OA and includes five questions about pain, two about stiffness, and 17 on degree of disability of activities of daily living.

The WOMAC pain scale ranges from 0 (no pain in all five activities: walking on flat surfaces, stair climbing, at night, sitting or lying down, and standing) to 20 (extreme pain in all five activities). The OAI protocol for WOMAC assessed pain in the past seven days. VAS scores for knee pain ranged from 0 (no pain) to 10 (the worst pain imaginable), with participants asked to rate their worst knee pain during the prior week.

All patients underwent MRI scans performed on a 3T MRI system (Trio; Siemens Medical Solutions, Germany). The full OAI pulse sequence protocol and sequence parameters have been published previously.^16^ To assess the cartilage damage, the femur and tibia were evaluated based on a division proposed by Eckstein and adopted in the OAI.^17^ This division focused on the loading surfaces of the medial and lateral femoral condyles, identified on the sagittal plane as the area between 60% and 75% of the distance from the trochlear notch to the posterior femoral condyle. The tibial plateau was divided into five regions, each representing different percentages of the total tibial cartilage area. The extent of cartilage loss was quantified as a percentage of subchondral bone denuded of cartilage in these regions for analysis purposes. The regions analyzed included the medial and lateral tibial hemiplateau, load-bearing areas of the medial and lateral tibial hemiplateau, and femoral condyles, among others. In this study, the femur and tibia were also divided into 15 anatomical portions for the BML evaluation using the MOAKS score.^15^ These portions were grouped into three subchondral bone regions: medial femorotibial, lateral femorotibial, and patellofemoral compartments. BML scores for each region were aggregated to calculate a total BML score. The Hoffa body synovitis score and meniscal extrusion (medial and lateral) were also evaluated based on the MOAKS system.

### Statistical analysis

All statistical analysis was performed using SPSS v. 19.0 (IBM, USA). All continuous data were expressed in terms of the mean (SD), while the categorical data were expressed as frequency and percentages. The Shapiro-Wilk test was to test normality of continuous variables. The Levene test was used to assess the homoscedasticity of the data. The Spearman rank correlation was used to assess correlations between pain (VAS, WOMAC, WOMAC pain) and BML score, age, BMI, and cartilage lesion areas. The Kendall’s tau correlation was used to assess associations between pain (VAS, WOMAC, WOMAC pain) and medial and lateral meniscus extrusion and Hoffa body synovitis. The influence of sex, of KL, the presence of patellar quadriceps tendinitis, the presence of effusion (bulge sign positive), the presence of anserine bursa tendernes, on pain (VAS, WOMAC, WOMAC pain) was assessed using one-way analysis of variance (ANOVA) when the scores were normally distributed and homoscedastic, the Mann-Whitney non-parametric test was used otherwise. The general linear model was used as multivariate analysis of the associations between total WOMAC pain score and scores of BML, articular cartilage, osteophytes, Hoffa synovitis, medial meniscus effusion and extrusion, and the partial eta square was reported. A p-value < 0.05 indicated statistical significance.

## Results

### Univariate analysis

**VAS:** The univariate analysis showed that VAS correlated with age (p = 0.011, ρ = −0.166), BMI (p = 0.006, ρ = 0.180), total % of exposed subchondral bone (p = 0.049, ρ = 0.129), total % of exposed subchondral bone of the femur (p = 0.014, ρ = 0.162), and had a trend of correlation with the % of exposed subchondral bone of the central medial femur (p = 0.082, ρ = 0.114) and with the % of exposed subchondral bone of the lateral compartment (p = 0.076, ρ = 0.117).

**WOMAC total score:** The univariate analysis showed that the WOMAC total score correlated with age (p = 0.037, ρ = −0.137), BMI (p = 0.003, ρ = 0.194), and had a trend of correlation with medial compartment BML (p = 0.118, ρ = 0.073).

**WOMAC pain subscale:** The univariate analysis showed that the WOMAC pain subscale correlated with BMI (p = 0.005, ρ = 0.185), and had a trend of correlation with age (p = 0.056, ρ = −0.125) and the extrusion of the medial meniscus (p = 0.096, *τ* = 0.086).

**Fig. 1:**
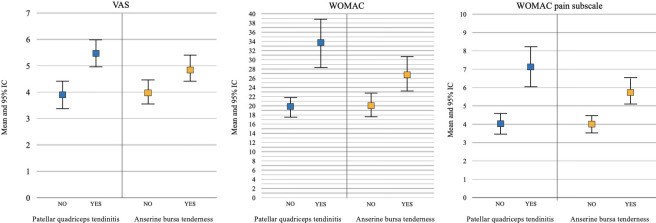
Univariate analysis results showing the correlation between visual analogue scale (VAS), Western Ontario and McMaster Universities Osteoarthritis Index (WOMAC) total score, and WOMAC pain subscale with patellar quadriceps tendinitis and anserine bursa tenderness.

The analysis also revealed how the presence of patellar quadriceps tendinitis and the presence of tenderness of the anserine bursa were associated with significantly higher values of VAS, WOMAC total score, and WOMAC pain subscale (p < 0.001 in all cases) (Figure 1). No other statistically significant correlations emerged between the clinical symptoms and the other parameters analyzed.

### Multivariate analysis

**Fig. 2:**
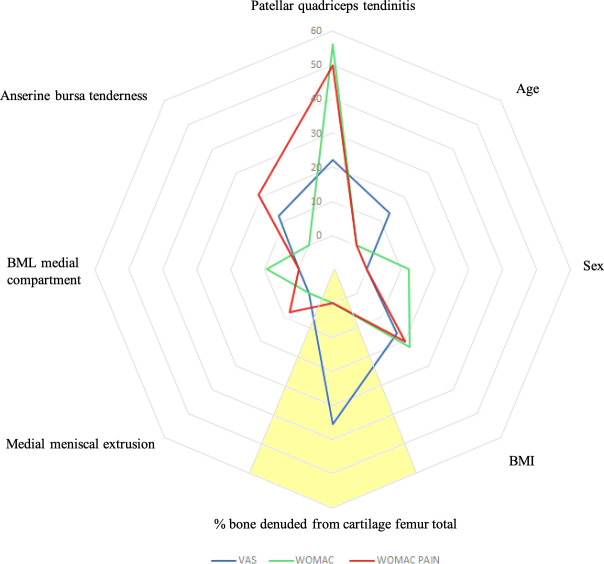
Graphical representation of the weight of the factors (in % of partial η^2^ values) influencing visual analogue scale (VAS) pain, Western Ontario and McMaster Universities Osteoarthritis Index (WOMAC) total score, and WOMAC pain subscale in patients with advanced knee osteoarthritis according to the multivariate analysis results. The yellow sector represents the cartilage degeneration domain and its correlation with symptoms. BML, bone marrow lesion.

A multivariate analysis was performed for VAS, WOMAC total score, and the WOMAC pain subscale (Figure 2). The analyses included all factors correlating with the clinical symptoms in the univariate analysis.

**VAS:** The multivariate analysis showed that the predominant factors influencing VAS pain were total % of bone denuded from cartilage in the femur (p < 0.001, partial η^2^ = 0.058), patellar quadriceps tendinitis (p = 0.004, partial η^2^ = 0.036), BMI (p = 0.013, partial η^2^ = 0.027), age (p = 0.026, partial η^2^ = 0.022), and anserine bursa tenderness (p = 0.033, partial η^2^ = 0.020).

**WOMAC total score:** The multivariate analysis showed that the predominant factors influencing the WOMAC total score were patellar quadriceps tendinitis (p < 0.001, partial η^2^ = 0.114), BMI (p = 0.001, partial η^2^ = 0.045), sex (p = 0.016, partial η^2^ = 0.025), and BML of the medial compartment (p = 0.035, partial η^2^ = 0.019).

**WOMAC pain subscale:** The multivariate analysis showed that the predominant factors influencing the WOMAC pain subscale were patellar quadriceps tendinitis (p < 0.001, partial η^2^ = 0.100), anserine bursa tenderness (p = 0.002, partial η^2^ = 0.040), BMI (p = 0.004, partial η^2^ = 0.036), and extrusion of the medial meniscus (p = 0.063, partial η^2^ = 0.015).

## Discussion

The main finding of this study is that, while influencing the pain level, the extent of cartilage degeneration is not the main factor influencing the overall symptoms experienced in advanced knee OA. Other intra- and extra-articular factors, including patellar quadriceps tendinitis, anserine bursa tenderness, BMLs, and BMI have a greater impact on pain and functional impairment of these patients.

The present study showed that the extent of cartilage damage plays a role, but it is not the main driver in determining pain symptoms in advanced knee OA, and it is not the primary factor contributing to functional limitation as well. This may be attributed to the nature of cartilage itself: as an aneural tissue, cartilage is not directly responsible for pain perception.^18^ However, its degeneration leads to the release of cartilage fragments and inflammatory mediators into the joint space, triggering synovial inflammation and affecting innervated structures, such as the joint capsule, subchondral bone, and periarticular tissues.^19^ This inflammatory process can result in the development and perpetuation of the pain symptomatology, representing a crucial factor determining this symptom. Conversely, functional impairment in knee OA appears to be more influenced by structural changes in the extra-articular components, such as tendons, bursae, and surrounding musculature, as well as biomechanical and patient-specific factors like BMI and age, which are less directly associated with cartilage damage.^20^

These results underscore the need for a comprehensive assessment of both intra- and extra-articular contributors when evaluating pain and functional impairment in advanced knee OA. The level of cartilage degeneration is often considered as a key aspect for treatment indication in the clinical practice and patient inclusion in OA trials. However, despite the pathogenic role of cartilage, there is evidence in the literature of individuals who, although presenting advanced stages of knee OA, remain asymptomatic, whereas others with even minor cartilage lesions experience significant functional impairment. A study by Son et al^7^ found that a considerable number of subjects with KL grade 4 OA did not report symptoms. In a study by Horga et al,^21^ knee MRIs of 230 healthy and asymptomatic subjects revealed the presence of numerous structural alterations, including cartilage damage, bone marrow oedema, and meniscal lesions. A study by Steenkamp et al^22^ showed that there was no correlation between functional scores, patient characteristics, and radiological severity of knee OA.

While the MRI assessment of cartilage degeneration may not be indicated in all cases in clinical practice, its use is of interest in the research setting, where it can shed some light on the status of the different tissues involved in the OA process. The lack of correlation between the radiological severity, such as the degree of cartilage degeneration, and the presence of functional impairment in advance knee OA suggests that symptom manifestation is not solely determined by cartilage damage. Instead, it is more likely to be influenced by multiple factors, including inflammatory mechanisms, biomechanical changes, and degenerative alterations in periarticular tissues. This aspect is of fundamental importance, particularly when selecting treatments for patients with advanced knee OA, where the most common solution is TKA to restore a joint at the end of the ‘wear and tear’ process. If the decision to proceed with surgery is guided primarily by the presence of pain and the presumed correlation with the severity of the disease, there is a risk of achieving unsatisfactory outcomes in terms of patient complacency. This may be one of the reasons explaining the considerable dissatisfaction rates of TKA, which in some studies exceed 20% of cases.^23^ This study demonstrated that the extra-articular structures represent the leading components influencing functional impairment in advanced knee OA, driven by pathological conditions of the knee extensor mechanism, such as patellar quadriceps tendinitis, as well as anserine bursitis.^24,25^ Failing to consider these determinants may lead to unsatisfactory results in solving the patient symptomatology.

Literature studies indicate that these factors may play a major role in a large percentage of these patients. Anserine bursitis is present in approximately 20% of individuals with knee OA and is positively associated with the severity of the disease and its symptomatology.^11^ Additionally, during acute exacerbations of knee OA, inflammation of the anserine bursa is one of the most frequently observed findings.^26^ Pathological alterations and inflammation of the extensor mechanism complex have also been highlighted as contributing to the onset of intense anterior knee pain, significantly impairing knee function and overall quality of life.^27^ Additionally, quadriceps strength was proven to be strongly associated with knee pain and disability.^28^ The predominant role of these extra-articular structures complicates the identification and understanding of the role of other intra-articular factors that may contribute to functional impairment.

Among these, the subchondral bone is considered one of the elements influencing knee pain, with multiple studies suggesting a correlation between the two.^29^ BMLs are a common finding in knee OA and are strongly associated with pain severity and disease progression.^30^ Various studies demonstrated that BMLs are linked to both weightbearing and non-weightbearing knee pain, with significant differences observed between individuals with painful knees and those without.^10^ BML size has been shown to correlate with the severity of knee OA pain, particularly during loading, suggesting a role in biomechanically induced pain in advanced OA stages, and BMLs are often accompanied by joint effusion, subchondral oedema, geodes, and reactive synovitis.^30^ Additionally, BMLs can rapidly change in size and are associated with cartilage degradation, pain exacerbation, and a poorer prognosis for disease progression.^31^ The findings of the present study put the previous research results in a broader context, highlighting the contribution of BMLs among the different factors influencing knee pain and functional impairment in advanced OA. In fact, BMLs were not the sole intra-articular component involved in advanced knee OA symptoms in this study.

Meniscal pathologies, particularly meniscal extrusion, also played a significant role. Menisci, being highly innervated structures, are crucial for axial load transmission and shock absorption.^32^ When meniscal extrusion occurs, it can lead to considerable knee pain. Furthermore, the loss of functional integrity linked to meniscal extrusion can result in the transfer of compressive forces to the cartilage and subchondral bone, intensifying the painful response. The findings of this study included the impact of meniscal extrusion on knee pain and functional impairment, highlighting its significant role among the key factors contributing to advanced knee OA symptoms. Moreover, not only intra- and extra-articular joint-related factors play a crucial role in the determination of pain and functional impairment in patients with advanced knee OA. Factors such as age and BMI are also relevant. A study by Rogers et al^33^ showed that among 576 individuals with knee OA, those with an elevated BMI were more likely to experience knee pain compared with those with a normal BMI, and this likelihood increased with each successive elevated BMI category. This multivariate analysis confirmed that BMI is a contributing factor influencing pain and function in patients with advanced knee OA.

These study findings hold significant clinical relevance, particularly in the management of patients with advanced knee OA. Identifying the primary drivers of pain and functional impairment can greatly influence treatment decisions, and through more targeted treatments improve patient outcomes. While femoral cartilage damage was an important factor contributing to the painful symptomatology, extra-articular structures, such as the quadriceps tendon, anserine bursa, and periarticular soft tissues, were shown to play a substantial role both in pain as well as in the functional limitation caused by advanced knee OA. When present, addressing these components is essential, especially in advanced OA patients who are often considered candidates for TKA.^23^ Misidentifying the source of pain and functional impairment, particularly when it stems from extra-articular structures or modifiable factors such as BMI, may lead to unnecessary surgeries and suboptimal results. This is of particular importance considering the rise of TKA surgeries and their risks, with the consequent impact on both patients and healthcare systems.^34,35^

An effective management strategy for advanced OA must prioritize a comprehensive assessment to distinguish between intra- and extra-articular pain contributors. For patients with predominant extra-articular involvement, conservative measures, such as targeted physiotherapy, are likely to be a more suitable treatment approach. Reviews consistently demonstrated the benefits of muscle strengthening and aerobic exercise in managing OA symptoms.^36^ Physiotherapy programmes focusing on improving joint range of motion, rebalancing periarticular muscle-tendon units, and strengthening the quadriceps and hamstrings can improve patellar tracking, reduce stress on the anserine, and alleviate pain.^37^ On the other hand, for intra-articular lesions, personalized interventions are required. Inflammatory components of the joint environment may benefit from topical and injectable therapies, which aim at restoring articular homeostasis.^38-41^ Finally, BMLs could be addressed through physiotherapy, bone-targeted pharmacological treatments, or surgical reinforcement of the subchondral bone, while meniscal extrusion may benefit from emerging centralization techniques as well as new substitution procedures.^42-44^

In light of these findings, invasive procedures such as TKA should be proposed only after a thorough evaluation to identify the leading triggers of pain and functional impairment. Recognizing the significant contribution of extra-articular structures, as well as modifiable factors like BMI, besides the cartilage degeneration level of the affected joint, can help avoid unnecessary surgeries, reduce failure rates, and improve patient satisfaction. A precise understanding of the multifactorial components of pain and functional impairment in advanced knee OA, emphasized by this study, can better inform surgical decision-making and facilitate more targeted treatment strategies.

This study has several limitations that should be acknowledged. First, the patient data were derived from a pre-existing database, and the analyzed outcomes were constrained by the information available in the OAI. Second, the retrospective nature of the study limits its strength. Third, the absence of information regarding whether patients were undergoing pain management, receiving knee treatments at the time of evaluation, or their specific functional demands introduces a potential bias. Despite these limitations, this study offers valuable findings regarding the primary drivers of knee OA symptoms, shedding light on the key factors contributing to pain and functional impairment in the advanced stages of this condition. Future research should focus on targeting the underlying causes of symptoms, guiding specific treatment strategies, and defining patient subgroups most likely to benefit from addressing each specific factor. Such efforts will enable clinicians to deliver personalized therapies and improve the overall management of knee OA in clinical practice.

In conclusion, the extent of cartilage degeneration influences the pain level, but is not the main factor influencing the overall symptoms experienced in advanced knee OA. Other intra- and extra-articular factors, including patellar quadriceps tendinitis, anserine bursa tenderness, BMLs, and BMI have a greater impact on pain and functional impairment of these patients, and should be considered when choosing the most suitable treatment approach to manage knee OA patients.

**Take home message**

- This study highlights that pain and functional impairment in advanced knee osteoarthritis are driven more by extra-articular structures, such as tendons, bursae, and subchondral bone, than by cartilage degeneration alone.

- Recognizing these contributors supports more accurate diagnosis and helps clinicians select targeted, individualized treatments beyond cartilage-focused or purely radiological decision-making.

## Data Availability

The data that support the findings for this study are available to other researchers from the corresponding author upon reasonable request.
